# Dietary Cholesterol Reduces Plasma Triacylglycerol in Apolipoprotein E-Null Mice: Suppression of Lipin-1 and -2 in the Glycerol-3-Phosphate Pathway

**DOI:** 10.1371/journal.pone.0022917

**Published:** 2011-08-09

**Authors:** Takashi Obama, Sayaka Nagaoka, Kazuki Akagi, Rina Kato, Naomi Horiuchi, Yasushi Horai, Toshihiro Aiuchi, Satoru Arata, Tomohiro Yamaguchi, Mitsuhiro Watanabe, Hiroyuki Itabe

**Affiliations:** 1 Department of Biological Chemistry, Showa University School of Pharmacy, Shinagawa, Tokyo, Japan; 2 Division of Molecular Metabolism and System Medicine, School of Medicine, Keio University, Shinjuku, Tokyo, Japan; 3 Center of Biotechnology, Showa University, Shinagawa, Tokyo, Japan; Clermont Université, France

## Abstract

**Background:**

Cholesterol metabolism is tightly regulated by both cholesterol and its metabolites in the mammalian liver, but the regulatory mechanism of triacylglycerol (TG) synthesis remains to be elucidated. Lipin, which catalyzes the conversion of phosphatidate to diacylglycerol, is a key enzyme involved in de novo TG synthesis in the liver via the glycerol-3-phosphate (G3P) pathway. However, the regulatory mechanisms for the expression of lipin in the liver are not well understood.

**Methodology/Principal Findings:**

Apolipoprotein E-knock out (apoE-KO) mice were fed a chow supplemented with 1.25% cholesterol (high-Chol diet). Cholesterol and bile acids were highly increased in the liver within a week. However, the amount of TG in very low-density lipoprotein (VLDL), but not in the liver, was reduced by 78%. The epididymal adipose tissue was almost eradicated in the long term. DNA microarray and real-time RT-PCR analyses revealed that the mRNA expression of all the genes in the G3P pathway in the liver was suppressed in the high-Chol diet apoE-KO mice. In particular, the mRNA and protein expression of lipin-1 and lipin-2 was markedly decreased, and peroxisome proliferator-activated receptor-γ coactivator-1α (PGC-1α), which up-regulates the transcription of lipin-1, was also suppressed. *In vitro* analysis using HepG2 cells revealed that the protein expression of lipin-2 was suppressed by treatment with taurocholic acid.

**Conclusions/Significance:**

These data using apoE-KO mice indicate that cholesterol and its metabolites are involved in regulating TG metabolism through a suppression of lipin-1 and lipin-2 in the liver. This research provides evidence for the mechanism of lipin expression in the liver.

## Introduction

A number of transcription factors are involved in the regulation of lipid metabolism in mammals. The expression levels of genes related to fatty acids and cholesterol (Chol) homeostasis are modulated by sterol regulatory element binding protein (SREBP) [1]. SREBP-1c regulates fatty acid metabolism, whereas Chol homeostasis is strictly regulated by SREBP-2 [2]. SREBP-2 is responsible for feedback regulation of the intracellular Chol concentration through expression of the LDL receptor and enzymes in the mevalonate pathway [3]. It is suggested that Chol metabolism and neutral lipid metabolism are interconnected, although the complete picture of neutral lipid metabolism remains to be established.

Triacylglycerol (TG) is synthesized via two pathways, the monoacylglycerol pathway and the glycerol-3-phosphate (G3P) pathway [4]. The former is predominantly found in the small intestine, while the latter is present in various tissues, including the liver. In the G3P pathway, G3P is acylated twice, by glycerophosphate acyltransferase (GPAT) and acylglycerophosphate acyltransferase (AGPAT). Then the resulting phosphatidic acid (PA) is dephosphorylated to generate diacylglycerol (DG) by the activity of PA phosphatase, and finally DG is acylated to produce TG by DG acyltransferase (DGAT). Recently, the genes responsible for the G3P pathway were identified. The lipin protein family consists of three isoforms named lipin-1, -2 and -3 in mammals, and was found to be the Mg^2+^-dependent PA phosphatase type 1 (PAP-1) enzyme that hydrolyzes PA to produce DG [5], [6]. Lipin-1, a human homolog of *fld*, which underlies lipodystrophy in the mouse [7], is expressed at high levels in white adipose tissue (WAT), skeletal muscle and testis, and is also detectable in the liver. In the liver, lipin-2 is more highly expressed than lipin-1 and functions as a major PAP-1 catalyst [8]. The expression level of lipin-3 gene is much lower in the liver [8]. The liver is the major organ of neutral lipid metabolism, and it is essential to clarify the regulatory mechanisms for de novo TG synthesis in the liver to understand the pathophysiology of metabolic diseases.

The apolipoprotein E-knockout (apoE-KO) mouse is widely used as a model animal for atherosclerosis accompanied by spontaneous hypercholesterolemia, due to the reduced clearance of VLDL and LDL from the circulation [9]. Hyperlipidemic conditions and atherosclerotic lesion development are accelerated when these animals are fed a high fat-high Chol diet [10]. It was also reported that treatment of apoE-KO mice with a diet supplemented with 1.25% Chol (high-Chol diet) increased the atherosclerotic lesion size in aortic tissue, as well as plaque vulnerability [11].

We found a profound decrease in the TG in VLDL fraction in apoE-KO mice fed a high-Chol diet. The regulatory mechanism of de novo TG synthesis has not been well reported in the literature. This led us to investigate regulatory mechanisms of TG synthesis in the liver that can be controlled by dietary Chol. We observed that numerous genes were influenced at the transcriptional level by the administration of the high-Chol diet, and found that Chol intake modulated hepatic TG synthesis through a significant suppression of lipin-1 and lipin-2. These data raise the possibility that Chol and its metabolites are factors which regulate TG metabolism in the liver.

## Materials and Methods

### Animals and diets

ApoE-KO mice (*apoe* ^−/−^:C57BL6), originally obtained from Charles River Laboratory, were bred in the Center for Laboratory Animal Science at Showa University. Mice were maintained in a humidity- and temperature-controlled room with a 12-h light-dark cycle. At 10 weeks of age, the mice were fed either normal chow (NIH formula; Oriental Yeast Co., Ltd) or the same chow containing 1.25% Chol (high-Chol diet) for either 1 week or up to 30 weeks. This study was approved by the Showa University Ethical Committee on Animal Experiments (approval ID 29041).

### Cell culture

HepG2 human hepatoma cells, purchased from RIKEN BioResource Center (Ibaraki, Japan), were maintained in DMEM medium supplemented with heat-inactivated 10% FBS, 100 IU/mL penicillin and 100 µg/mL streptomycin at 37°C and 5% CO_2_. To investigate the effects of bile acids, the cells (4.0×10^5^ cells/dish) were cultured in medium containing lipoprotein-deficient bovine serum (Sigma). The cells were homogenized and assayed by Western blotting, as described below.

### Plasma lipoproteins

The mice were anesthetized with ethyl ether, and blood was drawn from the inferior vena cava using EDTA as the anticoagulant. The blood was centrifuged at 1,000× g for 5 min to separate plasma. The chylomicron, VLDL and LDL fractions were separated by sequential ultracentrifugation using potassium bromide, followed by dialysis against PBS containing 0.25 mM EDTA [12].

### DNA microarray analysis

Microarray experiments were performed in three independent experiments at each diet. Total RNA was isolated from the liver of mice using an RNeasy kit (Qiagen). The quality of the extracted RNA fraction was evaluated using an Agilent 2100 Bioanalyzer (Agilent). DNA microarray analysis was performed by the one color labeling method using a Low RNA Input Linear Amplification Kit (Agilent) following the manufacturer's instructions. Briefly, a double-strand cDNA was synthesized by MMLV-RT using a primer corresponding to the T7 promoter. A cyanine-labeled cRNA was transcribed using T7 RNA polymerase, and then purified using the RNeasy mini kit. Yields and labeling efficiency were determined by ultraviolet absorption using a NanoDrop ND1000 spectrophotometer (Thermo Scientific). The labeled cRNA (500 ng) was fragmented at 60°C for 30 min with fragmentation solution, and then hybridized to the DNA microarray chip of the Whole Mouse Genome (4×44K) (Agilent) for 18 h at 60°C. After washing, the microarray chips were scanned with an Agilent microarray scanner. Individual microarray quality was evaluated using Feature Extraction software 9.1 (Agilent). Statistical analysis was performed using Gene Spring GX 7.3 software (Agilent) for normalizing and filtering. All microarray data is MIAME compliant and the raw and normalized data have been deposited in the MIAME compliant database Gene Expression Omnibus (GSE27457).

### Real-time RT-PCR analysis

cDNA was synthesized with a QuantiTect® Reverse Transcription kit (Qiagen) using 1 µg total RNA. Real-time RT-PCR analysis was performed using an ABI PRISM7000 (Applied Biosystems). The primers used are listed in Table S1. The PCR conditions were 2 min at 50°C and 10 min at 95°C, followed by 40 cycles of 15 sec at 95°C, 20 sec at 60°C and 40 sec at 72°C. The number of transcripts was quantified, and each sample was normalized on the basis of β-actin content.

**Table 1:** Bile acid concentration in the apoE-KO mouse liver.

Bile acid	Concentration (pmol/mg)	
	normal	high-Chol
Taurocholic acid	332.89	448.64
Taurodeoxycholic acid	21.60	31.98
Taurochenodeoxycholic acid	15.21	32.27
Tauroursodeoxycholic acid	10.79	14.71
Cholic acid	8.31	8.11
Ursodeoxycholic acid	2.19	2.56
Deoxycholic acid	0.85	0.26
Chenodeoxycholic acid	0.58	1.60
β-Muricholic acid	74.20	102.52
α-Muricholic acid	8.44	19.24
Tauro-α-Muricholic acid	16.82	36.79
Tauro-β-Muricholic acid	0.76	2.01
Glycocholic acid	0.77	0.58
Lithocholic acid	0.09	0.03
Taurolithocholic acid	0.15	0.66
Glycoursodeoxycholic acid	0.12	0.20
Glycochenodeoxycholic acid	0.01	N.D.
Glycolithocholic acid	N.D.	N.D.
Glycodeoxycholic acid	0.04	N.D.
Glyco-α-muricholic acid	N.D.	N.D.
Glyco-β-muricholic acid	N.D.	N.D.
**Total**	**493.82**	**702.16**

Small pieces of liver tissue (0.1 g) from three mice were combined, then the bile acids in the liver extracts were analyzed by HPLC, as reported previously [14]. N.D.: Not detected.

### Western blotting analysis

Approximately 50 mg of liver were homogenized in lysis buffer containing 50 mM Tris-HCl (pH 7.5), 250 mM sucrose, 1 mM EDTA, 1% protease inhibitor cocktail and 10 mM sodium orthovanadate, and then incubated on ice for 30 min, followed by filtration. The supernatant was collected and the protein concentrations were determined by BCA protein assay kit (Thermo Scientific) with BSA as the standard. An aliquot of the supernatant (30 µg protein) as well as HepG2 cell lysate was applied to 12% SDS-PAGE, then transferred to a polyvinylidene difluoride membrane (ATTO Co., Tokyo, Japan). The membrane was blocked with 0.1% Tween 20-TBS (TTBS) containing 2% skim milk and then incubated for 2 h with antibodies (Abs) against lipin-1 and lipin-2, respectively (R&D Systems, Inc.). The positive bands were visualized with a horseradish peroxidase-conjugated second Ab and ECL-plus Western blotting detection reagent (GE healthcare UK Ltd.), and detected using X-ray film (RX-U; Fuji Film Co.). The same membrane was reprobed with an Ab against β-actin (Santa Cruz Biotechnology, Inc.).

### Measurement of lipids

Chol and TG were extracted from the liver of mice by the method of Bligh and Dyer [13], and then dissolved in ethanol. The amounts of Chol and TG in the liver or in plasma were determined by enzymatic methods using the Total Chol-test Wako® and TG-test Wako® (Wako Pure Chemical Co., Osaka, Japan), respectively. Small pieces of liver tissue (0.1 g) from three mice were combined, then the bile acids in the liver extracts were analyzed by HPLC, as reported previously [14].

### Statistical analysis

Data are expressed as the mean ± SEM. Results were analyzed using Student's *t*-test, and statistical significance for all comparisons was assigned as * (*p*<0.05), ** (*p*<0.01), *** (*p*<0.005) or **** (*p*<0.001).

## Results

### Effect of a 1 week high-Chol diet on Chol and TG content in liver and plasma

**Figure 1:**
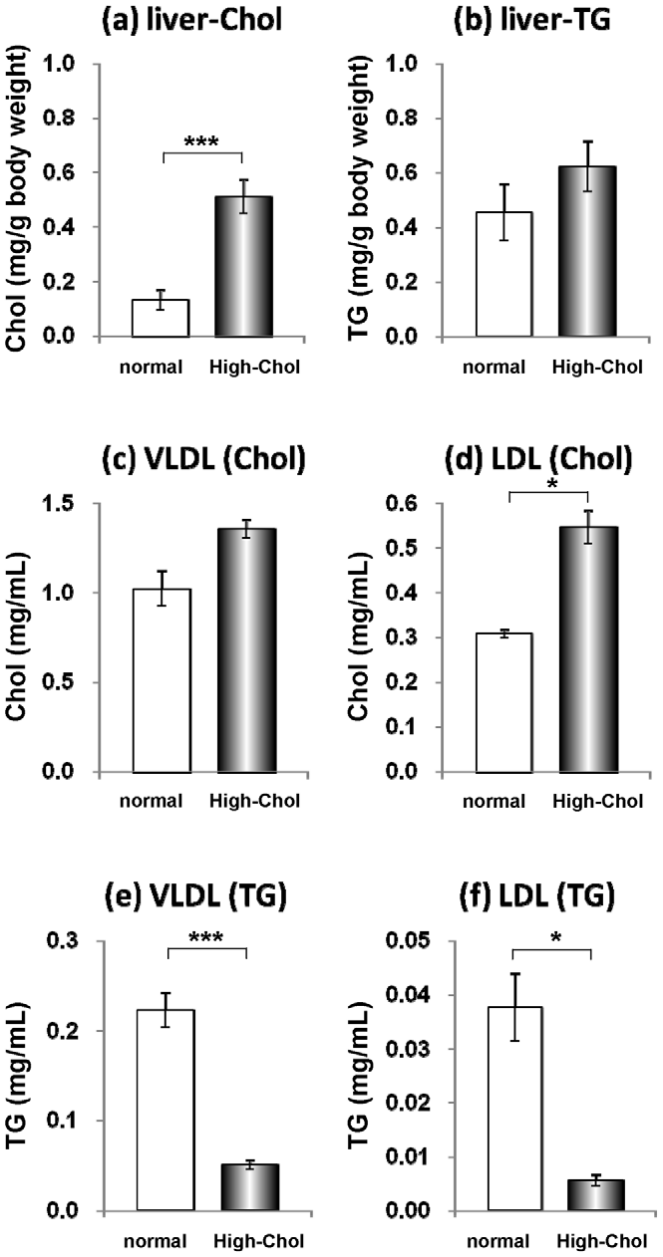
Accumulation of Chol in the liver and reduction of the plasma TG in the apoE-KO mice fed the high-Chol diet for 1 week. After apoE-KO mice were fed normal chow (white bar) or the high-Chol diet (black bar) for 1 week, the liver and plasma were collected (n = 3–4 for each group). (a, b) Chol (a) and TG (b) contents in the liver tissue were determined. (c–f) Lipoproteins in the plasma from the apoE-KO mice were fractionated by ultracentrifugation. Chol content in the VLDL (c) and LDL (d) fractions and TG contents in the VLDL (e) and LDL (f) fractions was determined. *, *p*<0.05; ***, *p*<0.005.

ApoE-KO mice are known as an animal model of hypercholesterolemia, in which the plasma concentrations of Chol are approximately 14-fold higher than in WT mice, even when maintained on normal chow (7.20±0.16 vs. 0.50±0.01 mg/mL). We examined the effect of dietary Chol intake on lipid metabolism in apoE-KO mice. ApoE-KO mice at 10 weeks of age were fed normal chow or the high-Chol diet (1.25% Chol) for one week. The amount of Chol in the liver increased 4-fold, but TG did not increase significantly (Fig. 1a, b). The high-Chol diet increased Chol in the plasma LDL fraction (Fig. 1c, d). However, the TG content in the VLDL and LDL fractions was reduced by 78% and 85%, respectively, compared with the normal chow group (Fig. 1e, f). These results suggest that VLDL-TG synthesis in the liver is suppressed in the apoE-deficient liver at an early stage of high-Chol diet treatment.

### Bile acid contents in the liver

Bile acids are the end products of hepatic Chol catabolism. We extracted bile acids from the mouse liver to perform analysis using HPLC. The total amount of bile acids in the liver increased by 40% in the high-Chol diet-treated mice compared with those receiving normal chow (Table 1). These data suggest that the apoE-KO mice fed the high-Chol diet accumulate both Chol and bile acids in the liver.

### Effect of long-term high-Chol diet on apoE-KO mice

**Figure 2:**
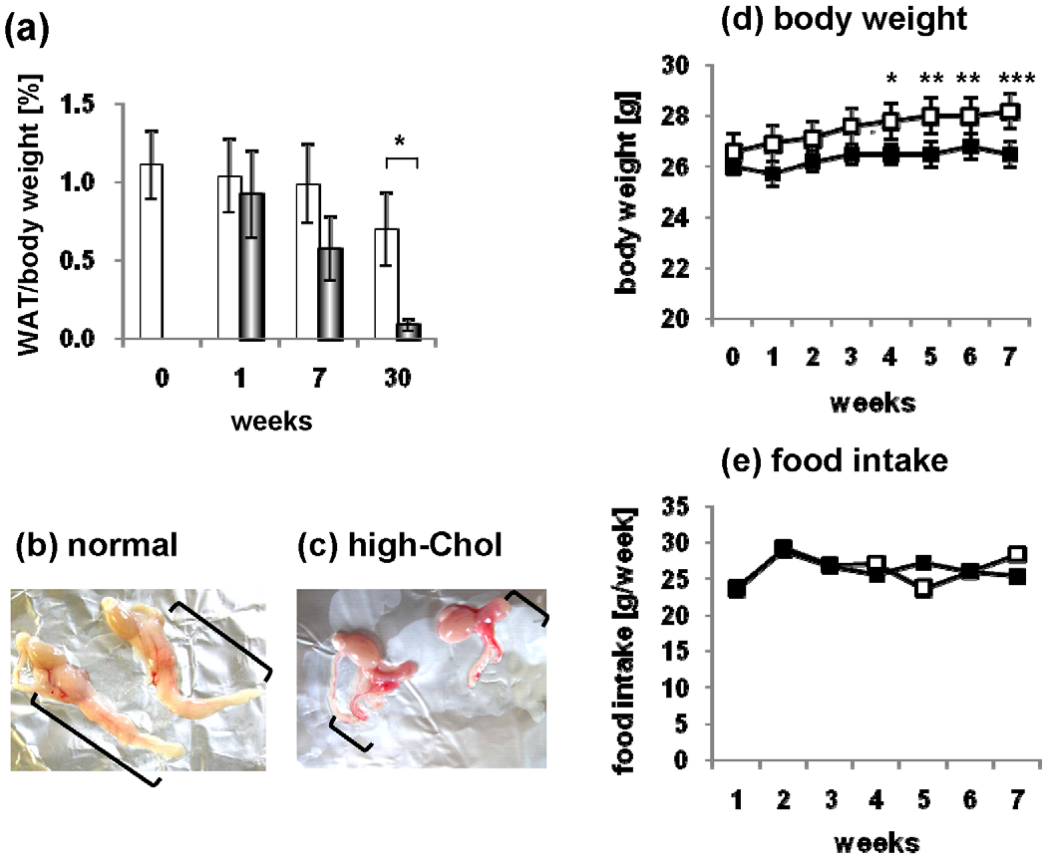
Effects of the high-Chol diet on apoE-KO mice. (a) The weights of the epididymal white adipose tissues (WAT) in apoE-KO mice fed normal chow (white bar) or the high-Chol diet (black bar) for up to 30 weeks were measured (n = 3–5 for each group). The data are expressed as the percent of epididymal WAT in the body weight (*p<0.05). (b,c) Epididymal tissues taken from mice fed normal chow (b) or the high-Chol (c) diet for 30 weeks. The brackets indicate the epididymal WAT. Note that WAT was diminished in the mice fed the high-Chol diet. (d) ApoE-KO mice (normal: n = 12, high-Chol: n = 14) were fed either the normal chow or high-Chol diet for up to 7 weeks. Statistical significance against the level at week 0 was calculated by ANOVA. *, *P<0.05*; **, *P<0.01*; ***, *P<0.005*. (e) The amounts of food consumed during each 7 day period in the cages were monitored (n = 2–4 for each cage, n = 10–12 mice in one group). The data are expressed as the amount of food consumed in a week per mouse.

ApoE-KO mice at 10 weeks of age were fed normal chow or the high-Chol diet for up to 30 weeks. The weight of epididymal WAT was gradually reduced by the high-Chol diet as early as 1 week, and then dramatically diminished over 30 weeks (Fig. 2a–c). The body weight of the apoE-KO mice fed normal chow increased significantly during the 7-week period, but not in those fed the high-Chol diet (Fig. 2d). These results were not due to changes in food intake, because food consumption was not reduced during the period (Fig. 2e).

### Influence of high-Chol diet on gene expression in the liver of apoE-KO mice

To verify the effect of the high-Chol diet on metabolism, we performed DNA microarray analysis of the liver from apoE-KO mice after 1 week of high-Chol diet feeding. A great many genes in the liver (up-regulated: 655 genes, down-regulated: 1,679 genes) changed their expression levels more than 1.5-fold under the high-Chol diet compared with normal chow. Table 2 shows the genes involved in lipid metabolism that exhibited changed mRNA expression levels as a result of the high-Chol diet. There was significant repression of sets of genes related to fatty acid biosynthesis, fatty acid metabolism, Chol biosynthesis, Chol homeostasis and TG biosynthesis.

**Table 2:** apoE-KO liver.

	Fold	*p* (n = 3)
cholesterol biosynthesis		
cytochrome P450, family 51	−5.56	<0.05
3-hydroxy-3-methylglutaryl-CoA reductase (HMG-CoAR)	−3.70	<0.01
hydroxysteroid (17-β) dehydrogenase 7	−2.27	<0.01
phenylalkylamine Ca2+ antagonist binding protein	−2.17	<0.05
farnesyl diphosphate synthetase	−2.08	<0.01
24-dehydrocholesterol reductase	−1.92	<0.05
lanosterol synthase	−3.10	<0.01
cholesterol homeostasis		
sterol O-acyltransferase 1 (ACAT)	1.90	<0.01
ATP-binding cassette, sub-family G (WHITE), member 1 (ABCG1)	2.00	<0.01
low density lipoprotein receptor (LDLR)	−1.85	<0.01
Transcriptional factor		
nuclear receptor subfamily 1, group H, member 4 (FXR)	−1.97	0.06
nuclear receptor subfamily 0, group B, member 2 (SHP)	2.03	0.15
hepatic nuclear factor 4, alpha (HNF-4α)	−1.42	0.11
peroxisome proliferator activated receptor alpha (PPARα)	−2.44	<0.05
retinoid X receptor alpha (RXRα)	−1.72	<0.05
forkhead box O1 (FOXO1)	−2.27	<0.05
bile acid transporter activity		
solute carrier organic anion transporter family, member 1a5	−1.57	<0.01
solute carrier family 10, member 2	−3.00	<0.01
bile acid biosynthesis		
cytochrome P450, family 7, subfamily a, polypeptide 1 (CYP7A1)	−3.36	0.10
cytochrome P450, family 39, subfamily a, polypeptide 1 (CYP39a1)	−1.65	0.15
cytochrome P450, family 8, subfamily b, polypeptide 1 (CYP8b1)	−2.86	0.15
fatty acid biosynthesis		
fatty acid synthase (FAS)	−2.12	0.16
fatty acid desaturase 1	−2.17	0.10
acyl-CoA synthetase medium-chain family member 3	−1.64	<0.05
elongation of very long chain fatty acids-like 2	−1.96	<0.05
thromboxane A synthase 1, platelet	2.00	<0.01
fatty acid metabolism		
acyl-CoA oxidase 2, branched chain	1.50	<0.05
carnitine palmitoyltransferase 1a, liver	−3.13	<0.05
acyl-CoA synthetase long-chain family member 1	−1.67	<0.01
acyl-CoA thioesterase 7	−1.59	<0.01
solute carrier family 27, member 2	−1.56	<0.01
acetyl-CoA acyltransferase 1A	−1.54	<0.01
lipid metabolism		
lipoprotein lipase (LPL)	2.80	<0.01
phospholipase A2, group VII	2.11	<0.01
hydroxysteroid 11-beta dehydrogenase 1 (11β-HSD1)	−1.67	<0.05
VLDL		
apolipoprotein B	−1.96	0.10
microsomal triglyceride transfer protein (MTP)	−1.64	0.12
TG biosynthesis		
acylglycerol-3-phosphate O-acyltransferase 6 (AGPAT6: GPAT4)	−1.61	<0.05
acylglycerol-3-phosphate O-acyltransferase 1 (AGPAT1)	−1.52	<0.01
lipin 1	−14.30	<0.05
lipin 2	−2.56	<0.05
diacylglycerol O-acyltransferase 2 (DGAT 2)	−1.92	<0.05

List of mRNA expression pattern in apoE-KO mice liver changed by high-Chol diet. The mice were fed with either normal chow or high-Chol diet (1.25% Chol) for 1 week. Cyanine-labbeled cRNA was prepared from total RNA extracted from the mouse liver tissue. DNA microarray analysis was performed as described in Materials and Methods. The affected genes (<1.5-fold) related to lipid metabolism are listed.

### Expression of the hepatic G3P pathway was down-regulated in the liver of high-Chol diet-treated apoE-KO mice

**Figure 3:**
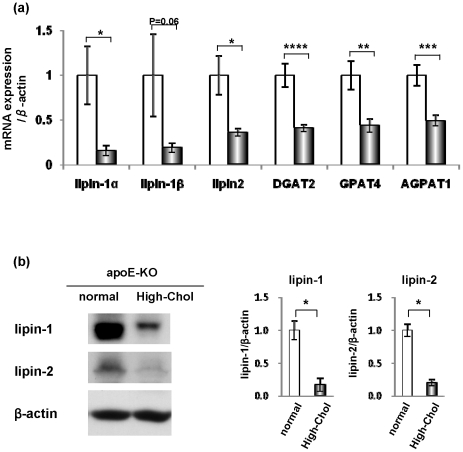
Reduction of the expression of genes and proteins involved in the G3P pathway in the liver. (a) The liver was collected from apoE-KO mice fed normal chow (white bar) or the high-Chol diet (black bar) for 1 week. The mRNA expression levels of genes involved in the G3P pathway (*lipin-1α*, *lipin-1β*, *lipin-2*, *DGAT2*, *GPAT4* and *AGPAT1*) were analyzed by real-time RT-PCR (n = 7). (b) Changes in the protein levels of lipin-1 and lipin-2 in the liver of apoE-KO mice fed normal chow or the high-Chol diet for 1 week were analyzed by Western blotting. Quantitative analysis is indicated in the graph (n = 3). *, *p*<0.05; **, *p*<0.01; ***, *p*<0.005; ****, *p*<0.001.

TG is synthesized via the G3P pathway in the liver. DNA microarray analysis revealed that the expression of genes in the G3P pathway, namely *GPAT*, *AGPAT1*, *lipin-1*, *lipin-2* and *DGAT2*, were reduced in the liver of the apoE-KO mice fed the high-Chol diet (Table 2). In particular, the *lipin-1* gene was the most repressed (14.3-fold) among all of the genes. The lipin-1 gene produces two transcripts, *lipin-1α* and *lipin-1β*, in the liver by alternative splicing [15]. The reduced mRNA levels for *lipin-1α* and *-2* were confirmed by real-time RT-PCR, although *lipin-1β* did not reach a significant difference (*p* = 0.06) (Fig. 3a). The mRNA levels of the other enzymes in the G3P pathway were also reduced significantly, as shown by real-time RT-PCR (Fig. 3a). Western blotting showed that the protein levels of both lipin-1 and lipin-2 were markedly decreased in the liver by the high-Chol diet (Fig. 3b). Note that the antibody against lipin-1 used binds to lipin-1α and lipin-1β since the antibody was raised against a domain conserved in the two isoforms. These data strongly suggest that TG synthesis was suppressed at the transcriptional level in the liver of apoE-KO mice fed the high-Chol diet.

### Reduction of the mRNA expression levels of transcription factors and genes involved in lipid metabolism

**Figure 4:**
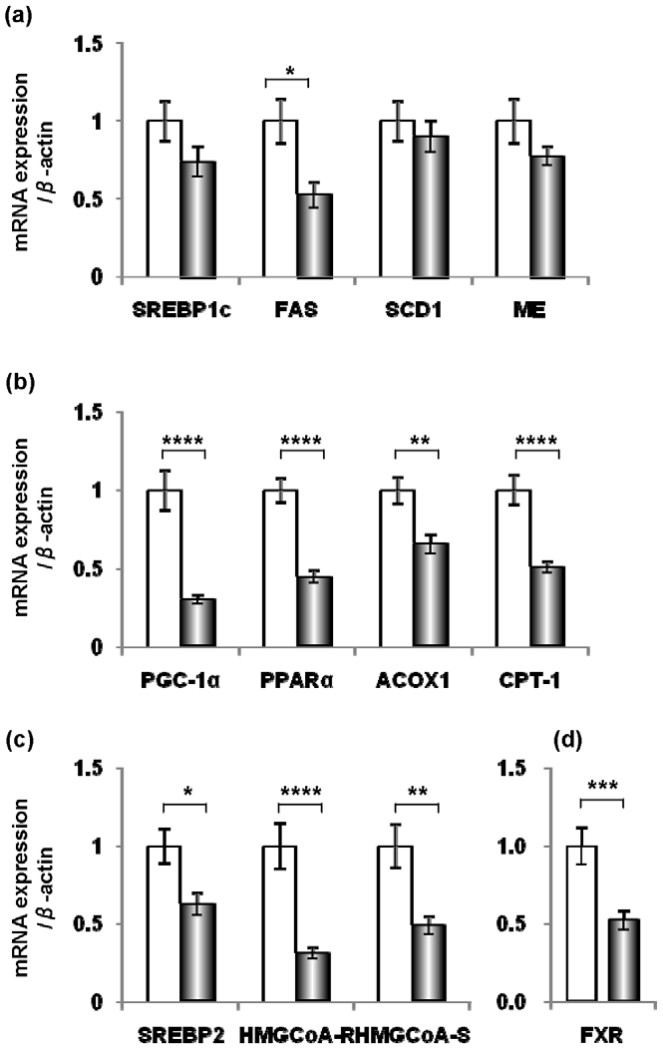
Hepatic mRNA expression of lipogenic genes in apoE-KO mice fed normal chow (white bar) or the high-Chol diet (black bar) for 1 week. The mRNA expression patterns of (a) *SREBP-1c*, *FAS*, *SCD-1* and *ME*, (b) *PGC-1α*, *PPARα* and its target genes (*ACOX-1* and *CPT-1*), (c) *SREBP-2* and its target genes (*HMG-CoA reductase* and *HMG-CoA synthase*) and (d) *FXR*, in the liver were analyzed by real-time RT-PCR (n = 7). *, *p*<0.05; **, *p*<0.01; ***, *p*<0.005; ****, *p*<0.001.

The effect of the high-Chol diet on mRNA expression levels of the lipogenic genes, *fatty acid synthase* (*FAS*), *stearoyl-CoA desaturase 1* (*SCD-1*) and *malic enzyme* (*ME*) was examined (Fig. 4a). The gene expression of *FAS* was significantly reduced in the mice fed the high-Chol diet, but *SCD-1* and *ME* were not. *SREBP-1c*, which regulates transcription of these lipogenic genes, decreased slightly, but did not reach statistical significance (Fig. 4a). These data suggest that the sterol-mediated regulation of fatty acid synthesis is not sensitive under this condition.

We then investigated the mRNA expression levels of other transcription factors that regulate lipid metabolism and energy homeostasis. As shown in Fig. 4b, the high-Chol diet suppressed the mRNA levels of *peroxisome proliferator-activated receptor (PPAR)- γ coactivator-1α* (*PGC-1α*) and *PPARα*. These results are in good accordance with the data from the DNA microarray analysis. To verify the significant reduction in PPARα, the mRNA expression patterns of PPARα target genes were analyzed. The mRNA levels of two genes, *acyl-coenzyme A oxidase 1* (*ACOX1*) and *carnitine palmitoyltransferase-1* (*CPT-1*), which are involved in β-oxidation, were reduced significantly (Fig. 4b). SREBP-2 and its target genes such as *HMG-CoA reductase* and *HMG-CoA synthase* were significantly down-regulated (Fig. 4c). The mRNA expression of *farnesoid X receptor* (*FXR*) was also suppressed (Fig. 4d). These data suggest that various transcription factors and enzymes related to lipid metabolism are suppressed in the liver of apoE-KO mice under the conditions of a high-Chol diet, although fatty acid metabolism via SREBP-1c is not affected.

### Effects of Chol metabolites on lipin expression

Chol is scarcely metabolized in peripheral tissues. The major metabolites of Chol are bile acids generated in the liver. In addition, certain specialized cells convert Chol into steroid hormones. We studied the possible involvement of Chol metabolites on the expression of lipin proteins. It is reported that the transcription of the lipin-1 gene is regulated by glucocorticoids (GCs) [16], [17] and by PGC-1α [18]. However, corticosterone, an active form of GC in mouse liver, did not change as a result of the high-Chol diet (Fig. S1). As shown in Fig. 4b, the high-Chol diet suppressed mRNA expression of PGC-1α in the liver of apoE-KO mice, suggesting that lipin-1 expression is down-regulated by a suppression of PGC-1α.

**Figure 5:**
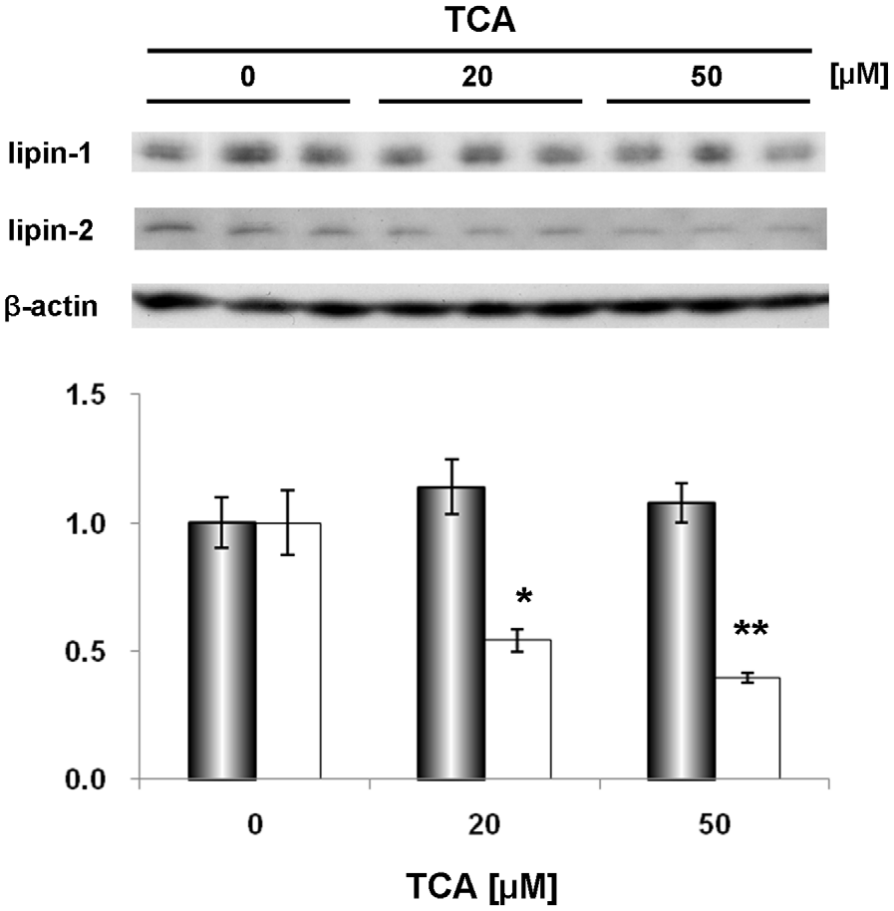
The effect of bile acids on lipin-1 and -2 protein expression in HepG2 cells. HepG2 cells were cultured in DMEM medium containing 10% lipoprotein-deficient serum , and TCA (0, 20, and 50 µM) was added for 24 h. Cells were lysed and analyzed by Western blotting. Quantitative analysis is shown with the graph (n = 3). *, *p*<0.05; **, *p*<0.01.

The regulatory system of lipin-2 expression is poorly understood. Although PGC-1α is a transcriptional regulator of lipin-1 gene, it is not involved in lipin-2 expression [19]. Since lipin-2 in addition to lipin-1 was down-regulated in the high-Chol diet condition, the effect of bile acids on lipin-2 expression was examined. When human hepatoma cell line HepG2 cells were treated with taurocholic acid (TCA), the lipin-2 protein level decreased, while the lipin-1 protein was not changed under the same conditions (Fig. 5). These results suggest that there is a bile acid-sensitive mechanism that regulates lipin-2 expression in the liver.

## Discussion

In this study we investigated the regulatory mechanisms of TG synthesis in the liver of apoE-KO mice fed a high-Chol diet, because we observed a dramatic reduction in TG content in the VLDL and LDL fractions under these conditions. These mice also showed a remarkable loss of epididymal WAT in the course of long term administration of the high-Chol diet. These observations led us to focus on the regulatory systems of TG metabolism in the liver. Genes related to lipid metabolism in the liver were suppressed, including the G3P pathway. Among them, the suppression of lipin-1 and -2 was remarkable, which may have reduced the TG content in lipoproteins. It is reported that lipin-2 is responsible for the major PA phosphatase activity [19] while hepatic lipin-1 has an important role in VLDL assembly and secretion in the liver [20], [21]. Our current result showing that lipin could have a significant impact on TG content in VLDL is in accordance with these studies. In addition, it is suggested that the accumulation of bile acids in the liver results in the suppression of lipin-2, which is responsible for TG synthesis.

Lipin consists of three genes, *lipin-1*, *-2* and *-3*, and the *lipin-1* gene produces three proteins, lipin-1α, -1β and -1γ by alternative splicing [6], [15], [22]. In adipocyte, lipin-1α localizes in the nucleus and lipin-1β localizes in the cytosol [15]. Several studies have reported that lipin-1 mRNA expression is induced by various mechanisms, including GCs [16], [17] and PGC-1α [18]. Our data suggest that a reduction of PGC-1α rather than GCs induces the suppression of lipin-1 expression by the high-Chol diet. Ishimoto *et al.* reported that human *lipin-1* mRNA and protein were changed by a depletion and supplementation of cellular sterols in HuH-7 cells in culture, and demonstrated that transcription of the *lipin-1* gene is mediated by SREBP-1 [23]. Our study showed strong reduction of lipin-1α and β protein concomitant with the mRNA expression of all the enzymes in G3P pathway occurs in vivo by dietary Chol. Although we cannot rule out the involvement of SREBP-1 in lipin-1 gene suppression, other regulatory molecules such as PGC-1α seem to be active under our conditions.

Recent studies reported that lipin-1 has a dual effect on TG synthesis and gene expression [18], [21]. Lipin-1 functions as a coactivator of PGC-1α and is an inducible amplifier of PPARα [18], and *lipin-1* mRNA levels were found to correlate with the *PPARα* mRNA expression level [24]. In this study, we found potent suppression of the *PPARα* mRNA level in the apoE-KO mice fed a high-Chol diet, accompanied by a suppression of its target genes such as *ACOX1* and *CPT-1*. These data are consistent with the recent report that neonatal *fld* mice, in which the *lipin-1* gene is mutated, exhibit a significant defect in fatty acid oxidation and hepatic steatosis [25]. SREBP-1c, another regulator of fatty acid metabolism, was not significantly changed in its expression pattern. These data strongly suggest that TG synthesis, in addition to Chol synthesis and fatty acid oxidation, was down-regulated in the liver of apoE-KO mice by Chol administration.

The regulatory mechanism of lipin-2 expression has not been reported. GCs specifically increased mRNA and protein levels of lipin-1, but not lipin-2 [16], [17]. Overexpression and deficiency of PGC-1α did not change the mRNA or protein levels of lipin-2 [19]. In this study, lipin-2 expression was decreased in the liver by a high-Chol diet and in HepG2 cells in the presence of bile acids, indicating a bile acid-dependent regulation of TG synthesis in the liver via lipin-2. This study is, to our knowledge, the first to show a direct link between lipin-2 down-regulation and bile acids. Recently, lipin-2 was reported to have an important role as the major PAP-1 enzyme in the liver responsible for glycerolipid synthesis. PAP-1 activity is significantly retained in the liver of adult *fld* mice, even though other tissues of the mice exhibit severely decreased PAP-1 activity [19]. RNAi suppression of lipin-2 markedly reduced PAP-1 activity in hepatocytes from both WT and *fld* mice, hence TG synthesis was suppressed despite the fact that fatty acid availability was high [19]. It is thus possible that (a) the suppression of both lipin-1 and lipin-2 in apoE-KO mice regulates TG metabolism through enzymatic and transcriptional pathways.

Since the amount of bile acids increased by 40% in the liver of apoE-KO mice fed the high-Chol diet, bile acid-dependent mechanisms may have led to the TG reduction in our experimental conditions. It was demonstrated that bile acids lowered TG synthesis via a reduction of the enzymes involved in fatty acid synthesis in the liver at a transcriptional level via activating FXR-SHP-SREBP-1c regulatory cascade. Bile acids activate FXR to induce SHP synthesis, and SHP suppresses the transcriptional function of SREBP-1c, leading to the suppression of lipogenic gene expression [26]. In this study, a significant reduction of *SREBP-1c* mRNA was not observed and the mRNA level of *SHP* did not change (data not shown), probably because the mRNA expression of *FXR* was suppressed by the high-Chol diet. Zhang et al. previously reported that PGC-1α increases FXR activity through two pathways, in which PGC-1α increases the *FXR* mRNA levels and interacts with FXR to enhance the transcription of FXR target genes [27]. In our experimental condition, the significant reduction of PGC-1α level may have led to a suppression of FXR.

Alternatively, it is reported that administration of bile acids reduced expression of PPARα-mediated genes, such as *ACOX1*, even in the FXR-null mice [28]. It seems that the suppression of PPARα functions may be significant under pathological, rather than physiological, conditions with increased bile acid concentrations. In our experiments we observed significant suppression of PPARα-mediated genes, *ACOX1* and *CPT-1*, after high-Chol diet treatment. Since the amounts of bile acids increased by 40% in the liver, bile acids might contribute to the changes in transcription profile of PPARα-mediated genes in the liver by Chol administration.

Stein et al. reported very recently that *apoE^−/−^ PGC-1α^−/−^* double knockout mice had reduced TG content in the VLDL fraction and a remarkable loss of adipose tissue weight [29]. The observation corresponds well with the current study, since the *PGC-1α* mRNA expression level was reduced in the liver by the high-Chol diet, and this supports the concept that PGC-1α has an important role in TG metabolism.

In conclusion, this study demonstrates that apoE-KO mice fed a high-Chol diet exhibit a significant reduction of plasma TG, accompanied by an accumulation of hepatic bile acid and suppressed expression of enzymes involved in the G3P pathway required for TG synthesis in the liver. We also found that bile acids have the ability to suppress lipin-2 expression in the liver. This study sheds new light on the mechanisms regulating TG synthesis and Chol metabolism as part of the context of whole-body neutral lipid homeostasis.

## Supporting Information

Figure S1 The inactive (dehydrocorticosterone) and active (corticosterone) forms of glucocorticoids in the liver of apoE-KO mice were analyzed by LC-MS/MS (n = 3).
(TIF)

Table S1 (DOC)
