# Supplementary figures and images for: Dietary Cholesterol Reduces Plasma Triacylglycerol in Apolipoprotein E-Null Mice: Suppression of Lipin-1 and -2 in the Glycerol-3-Phosphate Pathway
Source: PLoS One. 2011 Aug 9;6(8):e22917. doi: 10.1371/journal.pone.0022917 (PMC3153461; doi:10.1371/journal.pone.0022917)

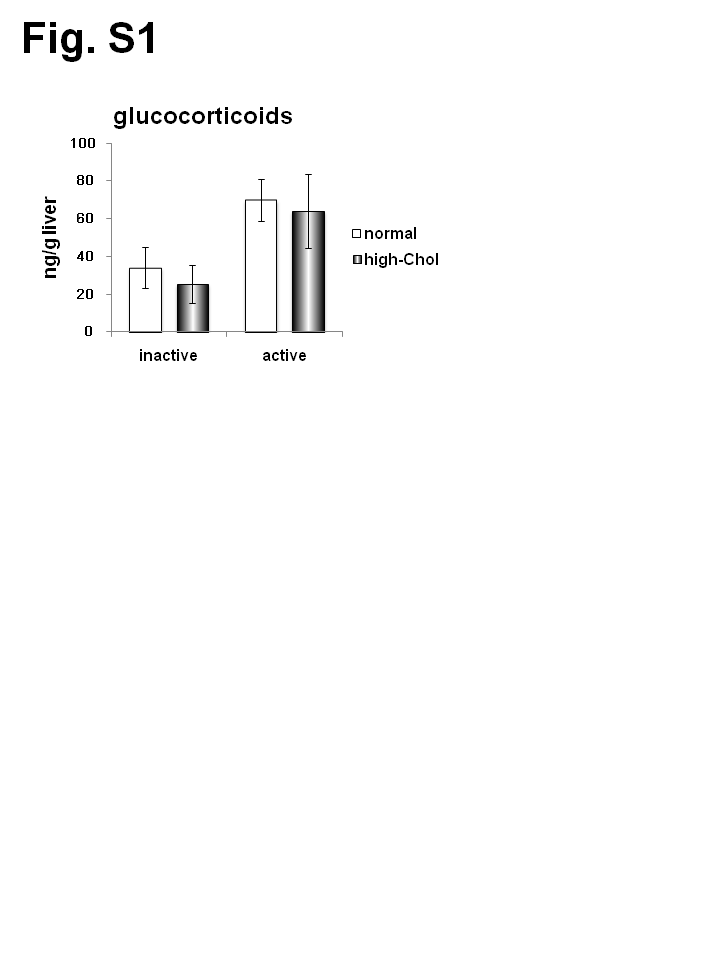

Supplement: Figure S1 — The inactive (dehydrocorticosterone) and active (corticosterone) forms of glucocorticoids in the liver of apoE-KO mice were analyzed by LC-MS/MS (n = 3). (TIF) [file pone.0022917.s001.tif]
