# Supplementary material for: Dietary Cholesterol Reduces Plasma Triacylglycerol in Apolipoprotein E-Null Mice: Suppression of Lipin-1 and -2 in the Glycerol-3-Phosphate Pathway
Source: PLoS One. 2011 Aug 9;6(8):e22917. doi: 10.1371/journal.pone.0022917 (PMC3153461; doi:10.1371/journal.pone.0022917)
Supplement: Table S1 — (DOC) [file pone.0022917.s002.doc]

**Table S1 Primers for real time RT-PCR**

| Genes | Forward | Reverse |
| --- | --- | --- |
| β-actin | ggccaggtcatcactattg | gaggtctttacggatgtcaac |
| ACOX1 | gcccaactgtgacttccatt | ggcatgtaacccgtagcact |
| AGPAT1 | cacccaggatgtgagagtctg | ctgacaacgtccaggcgagg |
| CPT-1  DGAT2 | gcactgcagctcgcacattacaa agtggcaatgctatcatcatcgt | ctcagacagtacctccttcaggaaa  aaggaataagtgggaaccagatca |
| FAS | gctgcggaaacttcaggaaat | agagacgtgtcactcctggactt |
| FXR | cttgatgtgctacaaaagctgtg | actctccaagacatcagcatctc |
| GPAT4 | ggcagaggagctggagtc | tgttgtggtacgtaatgatgg |
| HMG-CoA reductase | cttgtggaatgccttgtgattg | agccgaagcagcacatgat |
| HMG-CoA synthase | gccgtgaactgggtcgaa | gcatatatagcaatgtctcctgcaa |
| lipin-1α | ggtcccccagccccagtcctt | gcagcctgtggcaattca |
| lipin-1β | cagcctggtagattgccaga | gcagcctgtggcaattca |
| lipin-2 | agttgaccccatcaccgtag | cccaaagcatcagacttggt |
| ME | gccggctctatcctcctttg | tttgtatgcatcttgcacaatcttt |
| PGC-1α | aagtgtggaactctctggaactg | gggttatcttggttggctttatg |
| PPARα | cctgaacatcgagtgtcgaatat | ggtcttcttctgaatcttgcagct |
| SCD1  SREBP-1c  SREBP-2 | ccggagaccccttagatcga  ggagccatggattgcacatt  gcgttctggagaccatgga | tagcctgtaaaagatttctgcaaacc  gcttccagagaggaggccag  acaaagttgctctgaaaacaaatca |
